# Diversity Arrays Technology (DArT) Marker Platforms for Diversity Analysis and Linkage Mapping in a Complex Crop, the Octoploid Cultivated Strawberry (*Fragaria × ananassa*)

**DOI:** 10.1371/journal.pone.0144960

**Published:** 2015-12-16

**Authors:** José F. Sánchez-Sevilla, Aniko Horvath, Miguel A. Botella, Amèlia Gaston, Kevin Folta, Andrzej Kilian, Beatrice Denoyes, Iraida Amaya

**Affiliations:** 1 Instituto Andaluz de Investigación y Formación Agraria y Pesquera (IFAPA) Centro de Churriana, Cortijo de la Cruz, 29140, Málaga, Spain; 2 INRA, UMR 1332 BFP, F-33140 Villenave d’Ornon, France, Université de Bordeaux, UMR 1332 NFP, F-33140, Villenave d’Ornon, France; 3 Instituto de Hortofruticultura Subtropical y Mediterránea (IHSM-UMA-CSIC), Departamento de Biología Molecular y Bioquímica, Universidad de Málaga, 29071, Málaga, Spain; 4 University of Florida, Horticultural Sciences Department, Gainesville, Florida, 32611, United States of America; 5 Diversity Arrays Technology Pty Ltd, Building 3, University of Canberra, Bruce, ACT 2617, Australia; University of Guelph, CANADA

## Abstract

Cultivated strawberry (*Fragaria × ananassa*) is a genetically complex allo-octoploid crop with 28 pairs of chromosomes (2n = 8x = 56) for which a genome sequence is not yet available. The diploid *Fragaria vesca* is considered the donor species of one of the octoploid sub-genomes and its available genome sequence can be used as a reference for genomic studies. A wide number of strawberry cultivars are stored in *ex situ* germplasm collections world-wide but a number of previous studies have addressed the genetic diversity present within a limited number of these collections. Here, we report the development and application of two platforms based on the implementation of Diversity Array Technology (DArT) markers for high-throughput genotyping in strawberry. The first DArT microarray was used to evaluate the genetic diversity of 62 strawberry cultivars that represent a wide range of variation based on phenotype, geographical and temporal origin and pedigrees. A total of 603 DArT markers were used to evaluate the diversity and structure of the population and their cluster analyses revealed that these markers were highly efficient in classifying the accessions in groups based on historical, geographical and pedigree-based cues. The second DArTseq platform took benefit of the complexity reduction method optimized for strawberry and the development of next generation sequencing technologies. The strawberry DArTseq was used to generate a total of 9,386 SNP markers in the previously developed ‘232’ × ‘1392’ mapping population, of which, 4,242 high quality markers were further selected to saturate this map after several filtering steps. The high-throughput platforms here developed for genotyping strawberry will facilitate genome-wide characterizations of large accessions sets and complement other available options.

## Introduction

Efforts of crop improvement in polyploid species are hampered by the complexity of the genome and the difficulties to develop high-throughput genotyping platforms. Diversity Arrays Technology (DArT) offers an inexpensive and high throughput whole-genome genotyping technique as initially shown for rice [1]. The efficacy of DArT markers in the analysis of genetic diversity, population structure, association mapping and construction of linkage maps has been demonstrated for a variety of species, specially for plants (http://www.diversityarrays.com/dart-resources-papers). Furthermore, DArT has been applied successfully to species with large genomes such as barley [2] and with complex or/and polyploid genomes such as the decaploid sugarcane [3], hexaploid wheat and oat [4,5] or the paleoploid apple [6]. The DArT method allows for simultaneous detection of several thousand DNA polymorphisms (depending on the species) arising from single base changes and small insertions and deletions (InDels) by scoring the presence or absence of DNA fragments in genomic representations generated from genomic DNA samples through a process of complexity reduction [1]. Contrary to other existing SNP genotyping platforms, DArT platforms does not rely on previous sequence information. With the development of next generation sequencing (NGS), DArT technology faced a new development by combining the complexity reduction of the DArT method with NGS. This new technology named DArTseq™ represents a new implementation of sequencing of complexity reduced representations [7] and more recent applications of this concept on the next generation sequencing platforms [8,9]. DArTseq™ is rapidly gaining popularity as a preferred method of genotyping by sequencing [10–13]. Similarly to DArT methods based on hybridizations, the technology is optimized for each organism and application by selecting the most appropriate complexity reduction method (both the size of the representation and the fraction of a genome selected for assays) but was not yet applied in strawberry.

The genus *Fragaria*, which encompasses all soft-fruited strawberry species, belongs to the Rosaceae family, which comprises many economically important species such as apple, peach, and plum. *F*. *× ananassa* (2n = 8x = 56), the cultivated octoploid strawberry, is the most economically relevant soft berry, with a total harvested area of 361,662 ha and a production of 7,739,622 t in 2013 (FAOSTAT, 2015). In addition, strawberry is considered as a model species for the study of non-climacteric ripening in fleshy fruits and as so it is the subject of numerous studies [14,15]. This species resulted by a chance hybridization that took place in the early 1700s in a European garden between two related octoploid species, the North American *F*. *virginiana* and the South American domesticated *F*. *chiloensis* [16,17]. Systematic strawberry breeding began in Europe in the 1800s and shortly after in North America using a small number of the first European cultivars and native American clones [16]. As a result, genetic variability in this species has been shown to be limited, as only 53 founding clones (and only 17 cytoplasmic sources) were traced in the pedigrees of 134 North American cultivars [18,19]. Although a number of introgressions from wild octoploid species have later contributed to improved diversity of cultivated strawberry [17,20], breeding activities of the last decades focused on high-yielding cultivars with firm fruits have resulted in a dramatic loss of genetic diversity in modern cultivars [21,22].

In spite of its narrow genetic variation, strawberry shows a large diversity in many traits such as biotic and abiotic stress tolerance [23–25], fruit size, color, firmness and flavor [26–29]. In addition, different strawberry cultivars are well adapted to a large range of environments from tropical areas to the artic [30]. Using this natural variation for breeding better strawberries involves a long process of parental lines election, crosses and seedling selection that may take about 10 years [31]. The genetic characterization of strawberry accessions and the identification of polymorphic markers linked to important traits are key steps for the identification of appropriate parental lines and for increasing breeding efficiency through marker assisted selection (MAS).

Strawberry accessions have been genotyped using several methods such as Random Amplified Polymorphic DNA (RAPDs) [32,33], amplified fragment length polymorphisms (AFLPs) [32,34,35] or inter-simple sequence repeats (ISSRs) [36]. To date, the most used markers for assessing the genetic diversity as well as for genetic mapping in strawberry are microsatellites or single sequence repeats (SSR) markers due to a number of advantages such as reproducibility between laboratories [21,37–46]. Although SSRs can be multiplexed to some extent [40,43], none of the above systems are well suited for high-throughput genotyping, in contrast to single nucleotide polymorphisms (SNPs). However, the application of high-throughput SNP genotyping platforms has been delayed in polyploids in general and in the octoploid strawberry in particular and only recently have been developed for few species such as *Brassica napus*, wheat, sugarcane and cultivated strawberry [47–53]. The availability of a genome sequence for the diploid species *F*. *vesca* [54] allowed the development of the Axiom® IStraw90^®^ array, comprising more than 90K SNPs derived from short-read sequences from a panel of 19 octoploid accessions [52]. The diploid *F*. *vesca* reference genome displays high macrosynteny with the octoploid strawberries genomes [55], and particularly strong similarity to one of the 4 sub-genomes [56,57]. The usefulness of the IStraw90^®^ array for the genetic characterization of strawberry has already been shown [52,56]. However, the cost per sample is relatively high, making genotyping of large populations relatively expensive. Besides, SNP polymorphism relies on the relation of assayed accessions to those used in the construction of the array, limiting the usefulness when using more exotic populations [56]. These authors also noted that reliance on the *F*. *vesca* reference genome for the SNP discovery process has resulted in a bias towards markers in the *F*. *vesca*-derived sub-genome in comparison to the other 3 sub-genomes. An additional problem of the strawberry SNP array arises from interpretation of the complex signal dosages arising from the combination of alleles from the different sub-genomes [52].

To provide alternative high-throughput genotyping techniques useful for genetic analysis of diverse strawberry populations, here we report on the development of two DArT platforms for octoploid strawberry (DArT, http://www.diversityarrays.com), the second one taking benefit from the development of NGS. Our main objective was to prove DArT in a genetically complex species where several possible alleles were expected. The first DArT microarray platform was obtained from genomic representations derived from 62 widely diverse accessions that cover a wide range of variation based on phenotype, and geographical and temporal origin. Using this platform, we obtained a clear picture of the genetic diversity and structure of an octoploid strawberry collection. The second platform, DArTseq^TM^, thanks to NGS technologies, provided a much larger number of SNP markers compared to the DArT microarray and was successfully used to develop a high-density genetic map of strawberry using the ‘232’ × ‘1392’ population [42].

## Materials and Methods

### Plant material and DNA extraction

A total of 62 accessions of strawberry (*F*. × *ananassa*) were used for DArT marker development in this study, including the parental lines of the ‘232’ × ‘1392’ mapping population and 4 progenies. They were obtained from the IFAPA strawberry germplasm collection (ESP138) located at Centro IFAPA Churriana Málaga Spain or from the CIREF strawberry germplasm collection (FRA207) located at Douville France. Cultivar names, their year of release, pedigree and geographical origin are shown in Table 1. The chosen cultivars represent a wide range of variation based on agronomic traits, different geographical origins and pedigrees. The cultivars we studied were included in the European project GENBERRY collection and detailed information about each accession is publicly available at the European GENBERRY database (https://www.bordeaux.inra.fr/genberry/).

**Table 1:** List of *Fragaria × ananassa* germplasm used to evaluate the genetic diversity. The year of release, country of origin and pedigree is stated when available.

Accession	Date	Country	Pedigree
1392	2002	SPA	‘Gaviota’ × ‘Camarosa’
232	1996	SPA	Sel. 4–43 × ‘Vilanova’
93–04	2006	SPA	232 × 1392
93–54	2006	SPA	232 × 1392
93–85	2006	SPA	232 × 1392
93–88	2006	SPA	232 × 1392
‘Addie’	1982	ITA	‘Senga Pantagruella’ × MDUS 3816
‘Africa’	1870	EU	Unknown
‘Alaska Pionner’	1968	USA	‘Senga Sengana’ × ‘Alaska 292’ (*F*. *virginiana*)
‘Albion’	2004	USA	'Diamante' × Cal 94.16–1
‘Arking’	1981	USA	‘Cardinal’ × ARK 5431 (MDUS 3082 × ‘Delite’)
‘Betty’	2004	FRA	‘Pajaro’ × CF206
‘Camarosa’	1992	USA	‘Douglas’ × Cal 85.218 605
‘Candiss’	2008	FRA	CF1713 × 'Allstar'
‘Capitola’	1992	USA	CA75.121–101 × 'Parker'
‘Carisma’	1998	SPA	'Villanova' × 'Oso Grande' ('Parker' × CAL 77.3–603)
CF1116	1995	FRA	‘Pajaro’ × (‘Earlyglow’ × ‘Chandler’)
‘Capriss’ (CF3058)	2007	FRA	‘SweetCharlie’ × (‘Earlyglow’ × ‘Chandler’)
‘Rubis des Jardins’ (CF3453)	2009	FRA	‘Scott’ × ‘Chandler’
‘Charlotte’	2013	FRA	‘Mara des bois’ × Cal. 19
‘Ciflorette’	1998	FRA	‘Mamie’ × ‘Earliglow’
‘Cigaline’	1996	FRA	‘Garriguette’ × ‘Earliglow’
‘Cijosée’	1997	FRA	‘Mara des bois’ × Cal. 18
‘Cirafine’	2001	FRA	‘Mara des bois’ × Cal. 18
‘Darselect’	1996	FRA	‘Elsanta’ × ‘Parker’
‘Douglas’	1979	USA	(‘Tioga’ × ‘Sequoia’) × ‘Tufts’
‘Dover’	1980	USA	‘Florida Belle’ × USFL 71–189
‘Earlyglow’	1975	USA	MDUS 2359 (‘Fairland’ × ‘Midland’) × MDUS 2713 (‘Redglow’ × ‘Surecrop’)
‘Elsanta’	1981	NDL	‘Gorella’ × ‘Holiday’
‘Emily’	1995	GBR	‘Honeoye’ × ‘Gea’
‘Endurance’	2004	USA	PS-61 × PS-143
‘Fern’	1983	USA	‘Tufts’ × Cal. 69.62–103
‘Frau Mieze Schindler’	1933	DEU	‘Lucida perfecta’ × ‘Johannes Müller’
‘Fuentepina’	2009	SPA	NA-676 × SE-1-297 (‘Osogrande’ × ‘Carisma’)
‘Gariguette’	1972	FRA	(‘Pocahontas’ × ‘Regina’) × (‘Belrubi’ × ‘Marieva’)
‘Gento Nova’ (‘Nova Gento’)	2002	DEU	No data
‘Howard 17’	1909	USA	‘Crescent’ × ‘Howard 1’
‘Josif Mahomed’	-	RUS	Unknown
‘Jucunda’	1854	GBR	Old European selection of cultivated strawberry
‘Laxton's Noble’	1884	GBR	‘Sharpless’ × ‘Foreman's Excelsior’ (or reverse)
‘Little Scarlet’	1868	USA	Cultivar grown since the sixteen hundreds
‘Madame Moutot’	1906	FRA	‘Docteur Morere’ × ‘Royal Sovereign’
‘Mamie’	1990	FRA	‘Harvester’ × ‘Gariguette’
‘Mara des Bois’	1992	FRA	('Hummi Gento' × 'Ostara’) × ('Red Gauntlet' × 'Korona')
‘Medina’	1995	SPA	Z-45 × 'Parker'
‘Mysowka’	1958	RUS	No data
‘Nyoho’	1985	JPN	‘Kei 210’ × ‘Reiko’
‘Orléans’	2001	CAN	AC L'Acadie × ‘Jolie’
‘Pajaro’	1979	USA	‘Sequoia’ × Cal 63.7–101
‘Parker’	1983	USA	'Douglas' × Cal 71.98–604 (‘Tufts’ × Cal 63.7–101)
‘Frel’ (Pink Panda)	1991	USA	*F*. *× ananassa* × *Pontetilla Palustris* Hyb.
‘Rabunda’	1969	NDL	‘Redgauntlet’ × ‘Repita’
‘Saint Joseph’	1892	FRA	No data
‘Selva’	1983	USA	Cal 70.3–117 (‘zuster van Brighton’) × Cal 70.98–105 (‘Tufts’ × Cal 63.7–101)
‘Senga Sengana’	1954	DEU	‘Markee’ × ‘Sieger’
‘Sweet Charlie’ (‘Agathe’)	1997	USA	FL 80–456 × ‘Pajaro’
‘Tioga’	1964	USA	‘Lassen’ × Cal 42.8–16
‘Toyonoka’	1975	JPN	‘Himiko’ × ‘Harunoka’
‘Tribute’	1987	USA	EB18 × MDUS 42 58
‘Ventana’	1997	USA	Cal 93.170–606 × Cal 92.35–601
‘Vicomtesse H.’	1849	FRA	‘Elton’ × unknown
‘White Pine’	1860	-	Old cultivated strawberry with white fruits

The mapping population used to generate the octoploid strawberry map consisted of 94 F1 progeny lines derived from the cross between two heterozygous parents, ‘232’ and ‘1392’, with contrasting agronomical and fruit quality traits for which a linkage map was published previously [42,58].

Total genomic DNA from strawberry accessions was isolated from 130 mg of young unexpanded leaves using a modified CTAB method based on that of Doyle and Doyle [59]. DNA was quantified at 260 nm using a NanoDrop spectrophotometer (ND-1000 V3.5, NanoDrop Technologies, Inc.) and its quality was checked by two absorbance ratios, 260/230 and 260/280 nm, and by agarose gel electrophoresis. Two DArT platforms were developed using the 62 strawberry accessions as described in the next two sections.

### Development of the DArT microarray platform

The microarray-based DArT markers were developed by first testing eight combinations of the rare-cutting restriction enzyme PstI with different restriction endonucleases that cut frequently on DNA samples from the two parents and four progenies of the mapping population in order to identify the combination resulting in the most heterodispersed smear of restriction fragments (absence of any noticeable bands). The combination of PstI and TaqI produced most promising results and this complexity reduction method was applied to construct libraries of 7,680 genomic clones in total from 62 strawberry accessions (Table 1) as described [1]. In order to produce genomic representations, approximately 50 ng of genomic DNA was digested with PstI/TaqI combinations and the resulting fragments ligated to a PstI overhang compatible oligonucleotide adapter. A primer annealing to this adapter was used in PCR reaction to amplify genomic fragments and cloned into pCR2.1-TOPO vector (Invitrogen, Australia) as described previously [1]. The white colonies containing strawberry genomic fragments were picked into individual wells of 384-well microtiter plates filled with ampicillin/kanamycin-supplemented freezing medium [10]. Inserts from these clones were amplified using M13F and M13R primers in 384-plate format, PCR products dried, washed and dissolved in a spotting buffer. The amplification products were used as probes for printing DArT arrays on SuperChip poly-L-lysine slides (Thermo Scientific) using a MicroGrid arrayer (Genomics Solutions) and 7,680 cloned inserts (all printed in replication).

Each sample (the 62 diverse genotypes) was assayed using methods described above for library construction. Genomic representations were labeled with fluorescent dyes (Cy3 and Cy5). Labeled targets were then hybridized to printed DArT arrays for 16 hours at 62°C in a water bath. Slides were processed as described in [10] and scanned using Tecan LS300 scanner (Tecan Group Ltd, Männedorf, Switzerland) generating three images per array: one image scanned at 488 nm for reference signal measures the amount of DNA within the spot based on hybridization signal of FAM-labelled fragment of a TOPO vector multiple cloning site fragment and two images for “target” signal measurement. Signal intensities were extracted from images using DArTsoft 7.4.7 software (http://www.diversityarrays.com/software.html). DArTsoft was also used to convert signal intensities to presence/absence (binary) scores used in the downstream analysis. To determine marker quality (reproducibility of markers), 32 accessions were genotyped in technical replication (two independent libraries and marker extraction) and consistency of allele calling was used to determine reproducibility statistics and to select high-quality markers. In a polyploid like strawberry some of the missing data is due to a number of reasons such as copy number differences, presence of heterozygotes/hemizygotes or null alleles. The informativeness of the DArT markers was determined by calculating the polymorphism information content (PIC) within the 62 diverse strawberry cultivars [60]. The maximum PIC for dominant markers is 0.5. Both DArT assays and DArtsoft analysis were performed at DArT PL in Canberra, Australia.

### DArTseq Platform Development

Similarly to the DArT microarray, the DArTseq technology was optimized for strawberry by selecting the most appropriate complexity reduction method (both the size of the representation and the fraction of a genome selected for assays). Four methods of complexity reduction were tested in strawberry (data not presented) and the PstI-MseI method was selected. DNA samples are processed in digestion/ligation reactions principally as per [10] but replacing a single PstI-compatible adaptor with two different adaptors corresponding to two different Restriction Enzyme (RE) overhangs. The PstI-compatible adapter was designed to include Illumina flowcell attachment sequence, sequencing primer sequence and “staggered”, varying length barcode region, similar to the sequence reported previously [9]. Reverse adapter contained flowcell attachment region and MseI-compatible overhang sequence. Only “mixed fragments” (PstI-MseI) are effectively amplified in 30 rounds of PCR. The reaction conditions were 94°C for 1 min, followed by 30 cycles of 94°C for 20 sec, 58°C for 30 sec and 72°C for 45 sec, and then followed by a final extension step of 7 min at 72°C.

After PCR, equimolar amounts of amplification products from each sample were bulked and applied to c-Bot (Illumina) bridge PCR, followed by sequencing on Illumina GAIIx. The sequencing (single read) was run for 77 cycles in two lanes. Sequences generated were processed using proprietary DArT analytical pipelines. In the primary pipeline the fastq files are first processed to filter away poor quality sequences, applying more stringent selection criteria to the barcode region compared to the rest of the sequence. In that way the assignments of the sequences to specific samples carried in the “barcode split” step are very reliable. Approximately 600,000 (+/- 7%) sequences per barcode/sample were used in marker calling. Finally, identical sequences are collapsed into FASTQCOL. The propriety software package DArTsoft14 is used for marker discovery and scoring from FASTQCOL files. The FASTQCOL files from the samples of ‘232’ × ‘1392’ population were analyzed using DArTsoft14 to output candidate SNP and silicoDArT markers which are polymorphic within the set of samples (SilicoDArT markers are sequences with presence/absence variation in the DArTseq genomic representation). All unique sequences from the set of FASTQCOL files are identified, and clustered by sequence similarity at a distance threshold of 3 base variations. The sequence clusters are then parsed into SNP and silicoDArT markers utilizing a range of metadata parameters derived from the quantity and distribution of each sequence across all samples in the analysis.

Similarly to DArT microarray, a high level of technical replication is included in the DArTseq genotyping process, which enables reproducibility scores to be calculated for each candidate marker. The candidate markers output by DArTsoft14 are further filtered on the basis of the reproducibility values, average count for each sequence or row sum (sequencing depth), the balance of average counts for each SNP allele, and the call rate (proportion of samples for which the marker is scored).

### Statistical analysis of genetic relationships among accessions

DArTs were scored as 0/1 and they were used as different inputs for the RESTDIST and NEIGHBOR programs of the PHYLIP 3.6 software package to construct Neighbor-Join phylograms, based on Felsenstein’s modification of the Nei and Li restriction fragment distance [61]. Phylograms were rooted with 'Pink Panda' (hybrid between *F*. *× ananassa* and *Comarum palustre*, formerly *Potentilla palustris*). Clade strength was tested by 1,000 bootstrap analyses performed with the SEQBOOT program [62].

The genetic structure of the germplasm collection was analyzed performing Principal Coordinate Analysis (PCoA) implemented in the program GenAlex 6.41 [63] and by using STRUCTURE 2.1 software [64,65]. PCoA was based on standardized covariance of genetic distances calculated for DArTs markers. STRUCTURE software applies a Bayesian clustering algorithm to organize genetically similar individuals into clusters using multilocus genotype data. STRUCTURE sorts individuals into K clusters, according to their genetic similarity. The best K is chosen based on the estimated membership coefficients (Q) for each individual in each cluster. Twenty independent runs for K values ranging from 1 to 10 were performed with a burn-in length of 50,000 followed by 500,000 iterations. The admixture model was applied and no prior population information was used. The log-probability of the data, given for each value of K, was calculated and compared across the range of K. The software CLUMPP 1.1.2 [66] was used to find optimal alignments of independent runs and the output was used directly as input into a program for cluster visualization DISTRUCT 1.1 [67]. The optimal subpopulation model was investigated by considering ΔK, a second order rate change with respect to K, defined in [68], as implemented in STRUCTURE HARVESTER web page [69].

### Construction of the genetic linkage map

Selected SNP markers derived from the DArTseq platform were used in combination with previously mapped SSR, SSCP and AFLP [58] for map construction using JoinMap 4.1 [70]. Grouping was performed using independence LOD and the default settings in JoinMap and linkage groups were chosen from a LOD higher than 5 for all of them. Map construction was performed using the maximum likelihood (ML) mapping algorithm and the following parameters: Chain length 5,000, initial acceptance probability 0,250, cooling control parameter 0,001, stop after 30,000 chains without improvement, length of burn-in chain 10,000, number of Monte Carlo EM cycles 4, chain length per Monte Carlo EM cycle 2,000 and sampling period for recombination frequency matrix samples: 5. The integrated ‘232’ × ‘1392’ map was obtained using regression mapping and the ML-derived maps as starting order. The seven HGs were named I to VII, as the corresponding LGs in the diploid *F*. *vesca* reference map, followed by 1–4 (following the same order as in the previously published ‘232’ × ‘1392’ maps) for each of the 4 homeologous linkage groups. Linkage maps were drawn using MapChart 2.2 for Windows [71].

### Comparison between ‘232’ × ‘1392’ map and *F*. *vesca* genome

Physical map positions of DArT-derived SNPs and microsatellites used in this study were obtained by aligning the DArT sequences (Table A in S1 File) and SSR primer sequences to the most updated *F*. *vesca* pseudo-chromosome assembly [57] using Bowtie 2.1.0 [72]. For SSRs, we retained marker positions for those SSRs for which both forward and reverse primers mapped in paired-end alignment mode. For visualization of synteny, marker physical positions in mega-base pairs were multiplied by four to better fit the scale of the octoploid genetic maps in centimorgans (cM). Map comparisons were drawn using MapChart 2.2 for Windows [71].

## Results

### Genetic diversity

The set of 62 strawberry cultivars (see Material and Methods, Table 1) was characterized using 603 genome-wide DArT markers that proved to be polymorphic, showing the presence of low, intermediate and high frequency alleles. Although the 603 DArT markers were used in all the analyses, 247 presented at least one missing value while the remaining 356 were scored in all the accessions. The markers presented an average genotype call rate of 98.6% and an average scoring reproducibility of 99.71%. The average PIC value was 0.30, with only 20.4% of the markers having values lower than 0.10, 23.8% in the range of 0.1 to 0.30, 15.4% in the range 0.30 to 0.40, while the remaining 40.4% had PIC in the range 0.40 to 0.50. DArT markers in other species produced average PIC values such as 0.44 for wheat [73], 0.28 for sugar beet [74] or 0.21 for Lesquerella [12].

**Fig 1:**
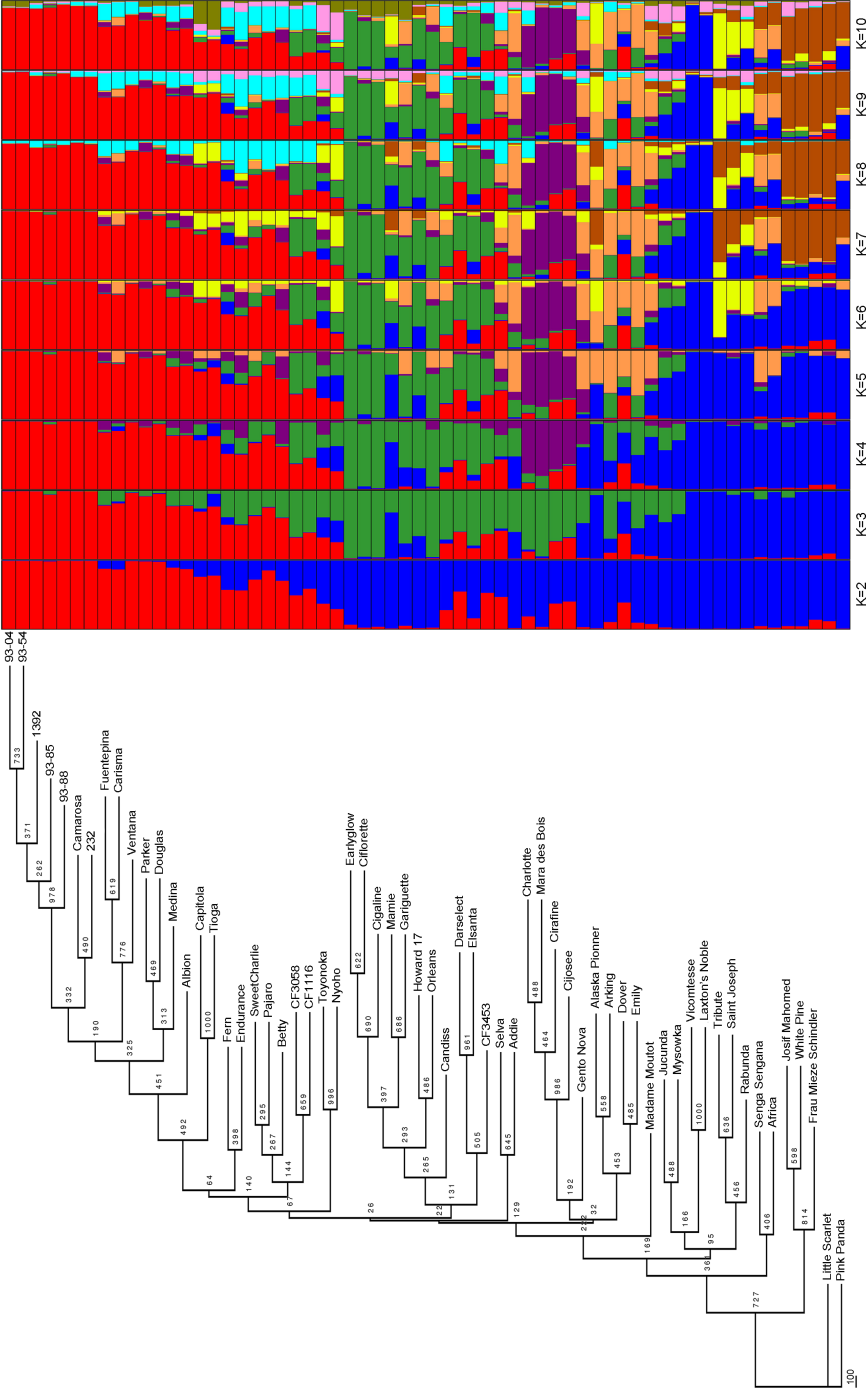
Cluster analysis of the strawberry collection based on 603 DArT markers. The Neighbor-Joining phylogram based on Felsenstein’s modification of the Nei and Li restriction fragment distance matrix using 'Pink Panda' (hybrid between *Fragaria × ananassa* and *Comarum palustre*) for rooting is shown on the left. Bootstrap values are shown on the branches. On the right, estimated population structure of the strawberry accessions using STRUCTURE. Genotypes are distributed in *K* = 2 to K = 10 ancestry groups. A horizontal bar represents each strawberry cultivar, and different colors quantify subgroup membership.

The Neighbor-Join Phylogram obtained with DArT markers produced several small clusters of related cultivars, and the majority of them contained cultivars sharing parental lines or close origin (Fig 1) validating the methodology. As examples, the Japanese cultivars ‘Nyoho’ and ‘Toyonoka’ were grouped, as occurred with ‘Parker’ and ‘Douglas’, ‘Carisma’ and ‘Fuentepina’ or ‘Darselect’ and ‘Elsanta’, all three pairs composed of a parent and a progeny (Table 1). The most diverse accession besides ‘Pink Panda’, used as outgroup, was ‘Little Scarlet’, which has been reported as a *F*. *virginiana* variety or a cross between *F*. *× ananassa* and *F*. *virginiana*. As shown in Fig 1, the phylogram derived from the DArT analysis reflects parental relationships between varieties and clearly clustered together those varieties bred for specific agro-climate areas and with a shared genetic background. This is evident for Californian/Mediterranean varieties such as ‘Douglas’, ‘Parker’ and derived accessions such as ‘Camarosa’, ‘Medina’, ‘Capitola, ‘Carisma’ and ‘Fuentepina. Similarly, the DArT-derived dendrogram resolved French accessions into two clusters: The first one comprised ‘Ciflorette’, ‘Cigaline’, ‘Mamie’ and their parental lines ‘Gariguette’ and ‘Earyglow’, and the second included ‘Mara de bois’ and derived cultivars ‘Charlotte’, ‘Cijosee’ and ‘Cirafine’ (Fig 1). Bootstrap support was moderate, with 20 nodes supported by bootstrap values higher that 50%.

### Population structure

The genetic structure of the strawberry accessions was analyzed using Principal Coordinate Analysis (PCoA) and the model-based Bayesian clustering method implemented in STRUCTURE. The most likely number of clusters (K) was evaluated considering the ΔK criterion [68], that gave the highest value at two groups, although an additional peak of ΔK was found also at K = 6. This method is known to give rise to the first structural level in the data and in the present study has led to discriminate strawberries varieties adapted to northern territories, many of them obtained previously to 1950, from those with Californian/Mediterranean pedigree, most of them obtained in recent years, represented by blue and red colors, respectively (Fig 1). The structure analysis using DArT markers was in agreement with the results displayed by the phylogram (Fig 1). A group of French cultivars including ‘Charlotte’ but also including the German ‘Gento Nova’ was separated as the purple subpopulation while the old European cultivars ‘Saint Joseph’ and ‘Rabunda’ shared admixture with the yellow subpopulation represented by ‘Tribute’. The remaining cultivars displayed different levels of admixture.

**Fig 2:**
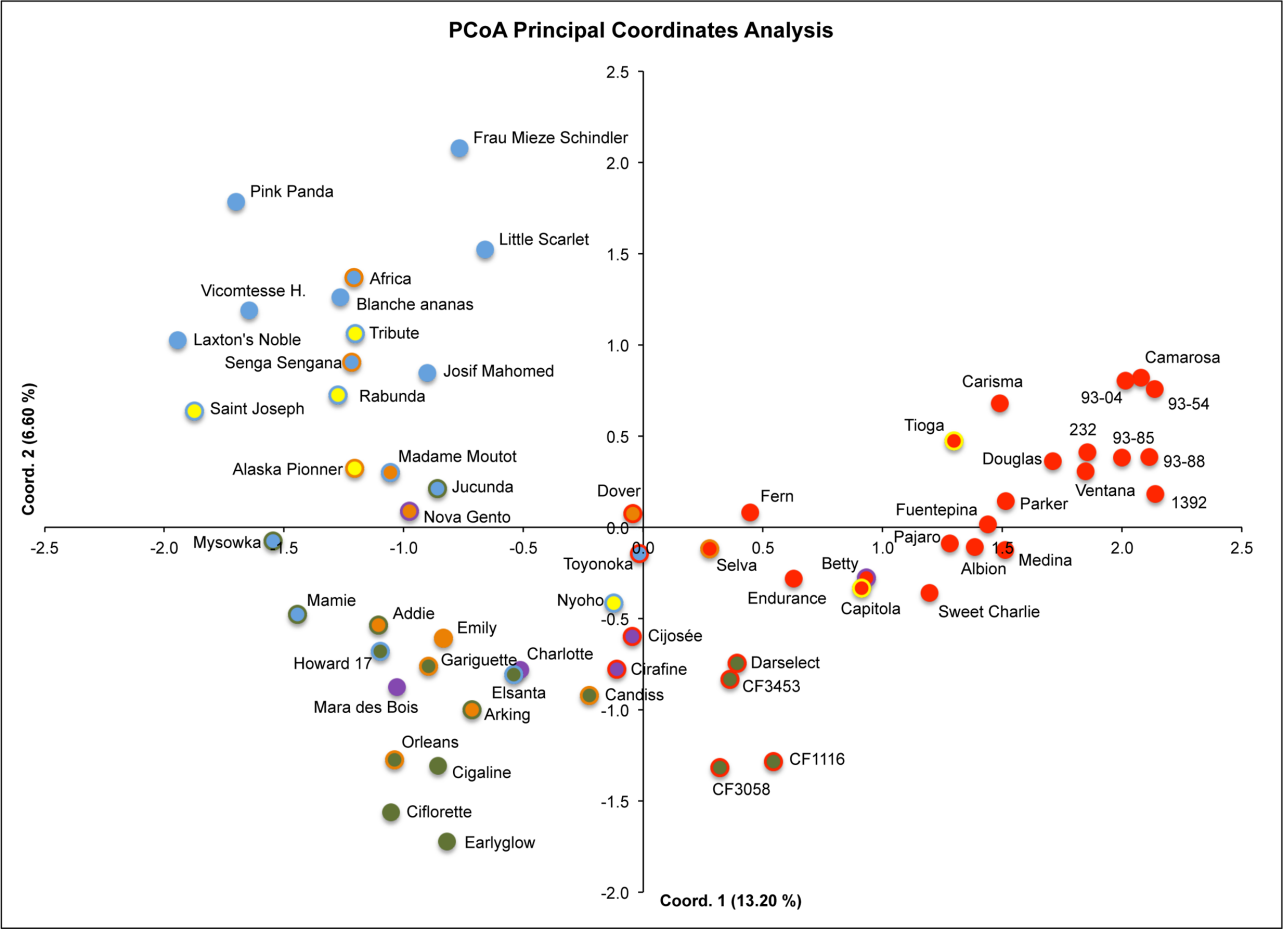
Principal coordinates analysis (PCoA) of 62 strawberry accessions based on 603 DArT markers. Accessions were labeled according to the STUCTURE results colors. Cultivars with admixed ancestry were labeled with the 2 most representing colors. The x axis represents the eigenvalue for principal coordinate 1 (PCo1) and the y axis for PCo2. The percentages of genetic diversity explained by the first and the second component were 13.20 and 6.06, respectively.

Genetic divergence among samples was also studied using DArT markers and the PCoA approach based on a genetic distance matrix with data standardization and it was largely consistent with the STRUCTURE results (Fig 2). The first axis explained 13,20% of variance and the second axis 6,06%. Using the same color code, both for STRUCTURE and PCoA, old European varieties, in blue, were located mainly in the first quadrant at the left; by contrast most recent varieties adapted to Mediterranean/Californian climate, in red, were located at the right quadrants. Increasing the number of structural levels additional parentage sources could be discriminated among the cultivars. Thus, French varieties in green were obtained from ‘Earlyglow’ or ‘Gariguette’, French varieties in purple derive from ‘Mara de Bois’, while the relationship among cultivars in orange and in yellow appears more obscure based in only the closest parental lines. The lack of additional pedigree data prevents us from further exploring their relationship (Fig 2).

### Genetic mapping

A total of 9,386 SNP markers were produced by the DArT platform and provided as 18,772 binary SNP allele scorings for the presence/absence (0/1) of the reference versus SNP allele scores. Due to the polyploidy of strawberry, DArTseq SNPs were filtered as alleles to avoid confusion between sub-genomes. A total of 6,744 (35.9%) of the SNP alleles was monomorphic in the progeny and were removed. Markers with missing values in more than 10% individuals (more than nine progeny lines) or in any of the two parental lines, or with 0 scores in both parents were excluded (1,551 alleles or 8.3%). The remaining markers (10,477 alleles or 55.8%) were tested for closeness to the various segregation ratios present in an octoploid species [35]. In the pseudo-testcross configuration and disomic inheritance, simplex markers are present in one parent and absent in the other or vice versa, and are expected to segregate 1:l (test-cross) in the F1 generation, while markers heterozygous in both parents are expected to segregate in a 3:1 ratio (inter-cross). Among the 10,477 markers, 3,014 (28.8%) fitted multiplex ratios (χ^2^ test; p = 0.01) and an additional 693 alleles (6.6%) did not fit the simplex ratios (both test-cross and inter-cross configuration; χ^2^ test; p = 0.001) and were regarded as distorted and also excluded. Among the remaining 6,770 simplex markers, 3,370 (49.8%) were in pseudo-test cross configurations (1,839 (27.2%) and 1,531 (22.6%) heterozygous in the female and male, respectively). The remaining 3,400 (50.2%) simplex markers were present in both parents and fitted a 3:1 ratio. The high number of 3:1 markers suggests a close relationship between the two parents, as previously reported [42], and shown by the Californian pedigree in Figs 1 and 2. The inter-cross markers are less informative compared to the test-cross markers and we therefore selected the most robust inter-cross markers by filtering 2,528 with row sums < 600 and kept only 872 out of the 3400 inter-cross markers.

The final number of selected SNPs was 4,242 (45.2% out of the 9,386 initial markers). Among them, 1,839 (43.3%) were ‘232’-derived markers, 1,531 (36.1%) were derived from ‘1392’ and 872 (20.6%) had an inter-cross configuration. The 4,242 SNPs were used for mapping, in combination with 408 SSR and gene specific markers previously mapped [58]. Only 194 SNP markers were excluded for being identical or loci with similarity >0.99, indicating low redundancy in the sequenced DArT clones. In general, identical loci were due to more than one SNP in the same DArT sequence. A total of 617 markers remained ungrouped after the grouping process in JoinMap 4.1. In order to increase the robustness of the linkage map and reduce the number of problematic markers, several additional markers were removed during the mapping process, either when they were positioned at less than 1 cM distance to another marker and/or displayed more than 5 genotypes with missing calls or when they generated high number of double crossover events distributed randomly on individuals. Therefore, these markers (despite they could be mapped) were discarded to optimize the linkage map for further QTL analyses in the future. For a number of SNP markers heterozygous in both parents (inter-cross), both SNP alleles were segregating as simplex markers (in the same sub-genome) and mapped to the same position of a LG. In those instances, we conserved only one of the two alleles in the map.

**Fig 3:**
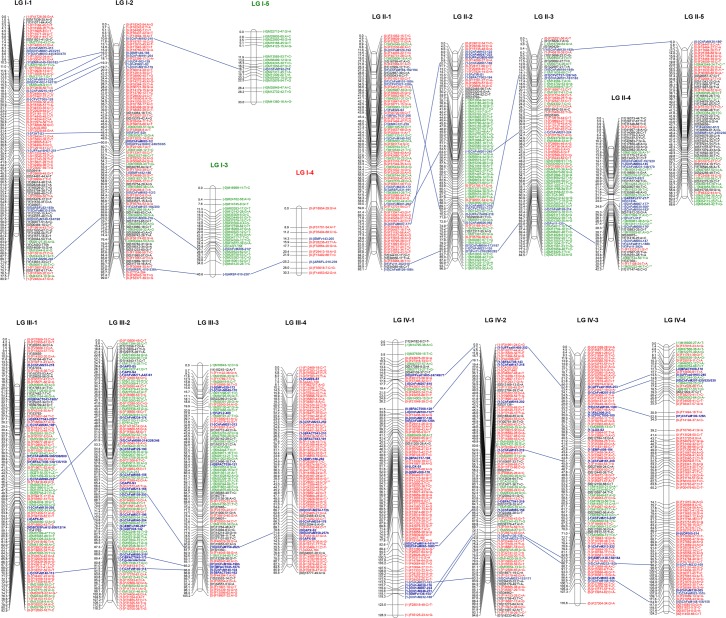
Integrated linkage map for strawberry using the ‘232’ × ‘1392’ population, DArTseq SNP and previously mapped SSR markers (Homoeology groups 1 to 4). Marker names and map distances are shown on the right and left side of each linkage group, respectively. Female and male-derived SNP markers are labeled in red and green, respectively. SNP markers heterozygous in both parents are in black while all SSR and gene specific markers are labeled in bold and blue. The name of each marker is preceded by their phases in each parental line.

**Fig 4:**
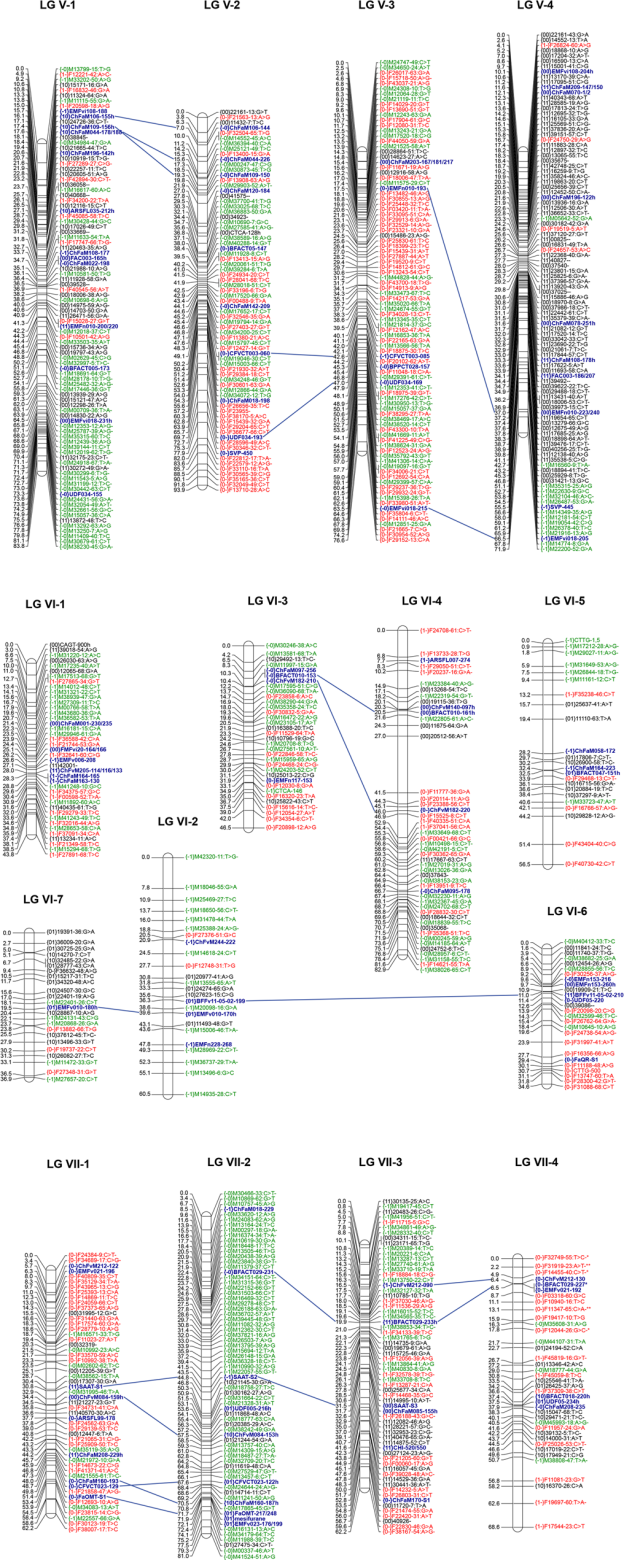
Integrated linkage map for strawberry using the ‘232’ × ‘1392’ population, DArTseq SNP and previously mapped SSR markers (Homoeology groups 5 to 7). Markers labeled as in Fig 3.

The final number of markers positioned in the consensus ‘232’ × ‘1392’ linkage map was 2,089 that provided high coverage of the genome as the 7 homoeology groups (HGs) were represented and the smallest LG was 30.3 cM long (Figs 3 and 4; Table B in S1 File). A total of 33 linkage groups (LG) were obtained that corresponded to the full complement of 28 strawberry chromosomes. LG I-4 contained only markers derived from ‘232’ and a number of LGs such as III-4, IV-1 or IV-4 were enriched in ‘232’-derived markers (Figs 3 and 4). Similarly, the maternal parent, ‘232’, may also have some large regions of homozygosity as two LGs (I-3 and I-5) contained only ‘1392’-derived markers and the majority of markers from LG VII-2 were also derived from ‘1392’. Markers were evenly distributed in the seven HGs, ranging from 220 markers in HG VI to 356 in HG IV and V (Table B in S1 File). For HGs III, IV, V and VII, the expected 4 LGs were produced and a similar number of markers was mapped across them (Table B in S1 File). For HGs I and II, one additional LG was obtained. In the case of homology group I, LGs I-3 and I-4 spanned only the lower half of the chromosome while LG I-5 spanned the top of the chromosome. A total of 7 linkage groups belonged to HG VI, with 4 of them being less than 50 cM long. The length of the ‘232’ × ‘1392’ map was 2,489.56 cM and the average distance between markers was 1.34 cM. Only 8 gaps were larger than 8 cM, with the largest gap of 14.5 cM located in the middle of LG VI-4. DArTseq SNPs were evenly distributed throughout the genome as they covered all and additional regions compared to the previously mapped SSRs (highlighted in blue in Figs 3 and 4).

### Comparison between the octoploid and the diploid reference genome

**Fig 5:**
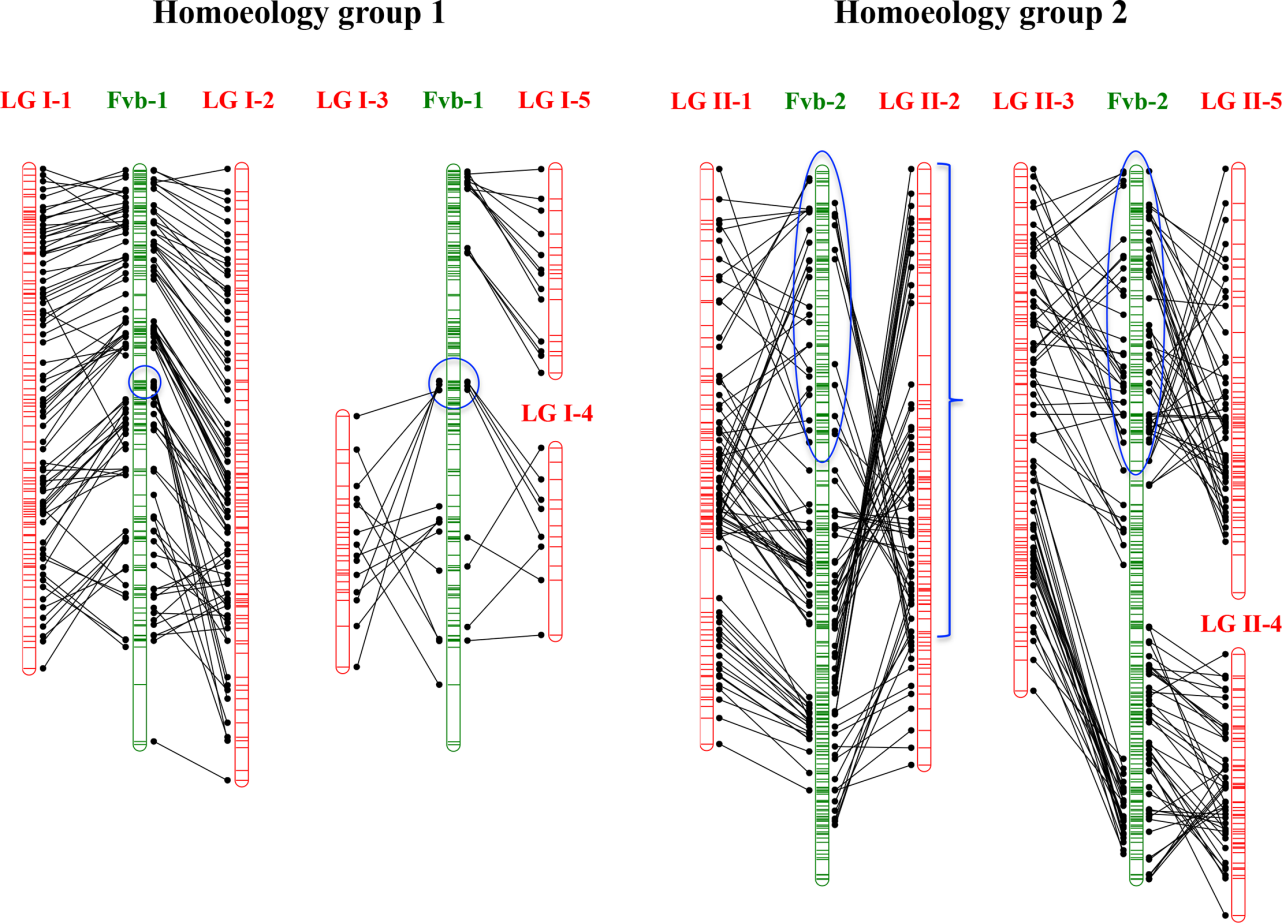
A comparison between diploid *Fragaria vesca* physical maps of chromosomes 1 and 2 (in green) and their LG homoeologues on the ‘232’ × ‘1392’ octoploid linkage map (in red). Marker order in *F*. *vesca* is based in the genome assembly of Tennessen et al., 2014. Rearrangements are highlighted in blue.

Out of the total 2,089 mapped markers, only 79 markers (3.8%) were mapped on a different chromosome to that expected based on the latest assembly of *F*. *vesca* genome [57] (Table A in S1 File). This supports that macrosynteny is conserved between these two species with only a limited number of interchromosome rearrangements, as previously reported [46,57,75,76]. Although overall marker order was conserved between the developed octoploid map and the reference genome, intrachromosome rearrangements were abundant (Fig 5; S1 Fig). Many of these rearrangements were conserved in more than one homoeologous LG such as one detected in the middle of pseudochromosome 1 and the lower part of three *F*. *× ananassa* LGs belonging to HG I, an inversion in a segment at the top of pseudochromosome 2 in comparison to three *F*. *× ananassa* LGs of HG II or another in *F*. *vesca* pseudochromosome 3 and three homoeologous LGs in *F*. *× ananassa* HG III. In other instances, rearrangements were detected in only one homoeologous LG compared to *F*. *vesca* or the rest of the sub-genomes, as one large inversion involving more than half of LG II-2 (Fig 5; S1 Fig). Another type of discrepancy between the ‘232’ × ‘1392’ map and the *F*. *vesca* physical map involved mostly single loci that showed large differences in their position. Examples include those detected in HG VI and VII (S1 Fig).

## Discussion

### DArT platforms provide reliable high-throughput genome-wide analyses in the cultivated octoploid strawberry

Our study highlights the power of the strawberry DArT platforms to provide novel insights into the genetic architecture of the genetically complex octoploid strawberry, *F*. *x ananassa*. They provide robust information of hundreds to thousands of markers across the octoploid genome without the requirement of a sequenced reference genome.

Compared to the DArT microarray platform, which is based on genome complexity reduction using restriction enzymes followed by hybridization to microarrays [1], the DArTseq^TM^ platform [10,77] combines the DArT platform with NGS sequencing, providing higher number of markers and offering the opportunity to anchor the markers on the reference genome of the diploid woody strawberry *F*. *vesca* [57] (Figshare: http://dx.doi.org/10.6084/m9.figshare.1259206). In molecular breeding, this advantage is important for developing new markers for marker-assisted selection based in the identified DArT marker sequences. The DArT clones used to analyze diversity in strawberry could be sequenced for future works or for comparison to the mapped DArTseq^TM^ markers. However, the choice of complexity reduction method was optimized to generate the optimal restriction fragment size for each platform and would result in a very small overlap of markers between them. Furthermore, the higher cost-effectiveness and larger number of markers generated by the DArTseq^TM^ platform makes this technology more useful for future studies.

SSRs have been the preferred marker for genetic diversity as well as for QTL mapping in strawberry [21,37–46]. To overcome the limited number of SSR markers, recently, a database listing a high number of SSRs in the cultivated strawberry was reported [45] (http://marker.kazusa.or.jp/strawberry/). However, high throughput platforms offer the advantage of cost and time efficient whole genome coverage. After this work, two complementary platforms are now available for high throughput genotyping of the octoploid strawberry: the DArTseq here developed and the 90K Axiom^®^ SNP array [52]. The first one offers a cost-effective genotyping approach, yielding a large number of markers with easy interpretation as dominant markers. The DArTseq derived SNP markers can alternatively been used as codominant markers. However, caution should be taken that both the reference and the SNP segregate as single dose markers in the same sub-genome. Genetic mapping of DArT markers have resulted in a remarkably homogeneous distribution across the genome (Figs 3 and 4). In addition, previous studies have shown that the use of PstI, a methylation-sensitive restriction enzyme, in PstI-based DArT markers predominantly targets low-copy, gene-rich regions of the genome [11,78,79]. Furthermore, the mapped DArTseq SNPs did not show a preferential distribution to one of the sub-genomes of octoploid strawberry. In comparison to DArTseq^TM^ and other genotyping by sequencing approaches, practically all fixed arrays suffer from ascertainment bias, especially when developed using not very representative reference genome and fairly small sampling of diversity for marker discovery. In the particular case of the 90K Axiom^®^ SNP array developed for strawberry, it was based on the *F*. *vesca* reference genome and, when used for mapping in the octoploid strawberry, suffers from a bias to one of the sub-genomes, as shown in the ‘Holiday’ × ‘Korona’ and DA × MO linkage maps [52,56]. Therefore, the strawberry DArTSeq^TM^ pipeline can be used as an useful alternative to fixed sequence approaches for molecular diversity analyses and to generate extremely dense linkage maps suitable for QTL detection and genome-wide association studies (GWAS).

### Structure of the genetic diversity highlights the history of strawberry breeding

The analysis of genetic diversity and population structure here reported highlights the history of the two first centuries of the cultivated strawberry breeding programs, which have been conducted in the past mainly in USA and Europe. Breeding of the cultivated strawberry begun shortly after its origin in the 1760s, when a cross between the Scarlet strawberry (*F*. *virginiana*) as pollen source, and the ‘Frutilla’ or Chilean strawberry (*F*. *chiloensis*) occurred accidentally [16]. First breeding work was conducted in the middle of the 1800s, mainly in England and in North America, and following this period, new cultivars were introduced in Europe where breeding efforts intensified at the end of the Nineteenth century [31].

As shown in Fig 1, cluster analysis of the varieties using the DArT markers reflects these relationships in breeding programs. Although bootstrap support values were in general low, and therefore the reliability of several branches low, the results obtained using DArT markers are highly in agreement with previous reports [21,22,40]. A first group is organized around the very active breeding programs during 1960s – 1970s in California [80] leading to cultivars such as ‘Parker’, ‘Douglas’, ‘Pajaro’ or ‘Fern’, and more recently ‘Camarosa’. After their introduction in Europe, new cultivars well adapted to Mediterranean countries such as ‘Medina’ or ‘Carisma’ were selected in Spanish breeding programs using Californian parents. A second group including genotypes organized around ‘Darselect’, ‘Elsanta’, ‘Earlyglow’ and the old USA founder ‘Howard 17’ gathered old USA cultivars with European cultivars selected at the end of the twentieth century. The last group included genotypes belonging to old European varieties, e.g. ‘Saint Joseph’, ‘Vicomtesse’, ‘Josif Mahomed’, ‘Mieze Schindler’ and ‘Jucunda’. This group was also clearly observed in a previous analysis of strawberry genetic diversity [22]. These results suggest that old European breeding programs led to lines showing different alleles than those selected today. In addition, the wide dispersion of this group in the PCoA (Fig 2) compared to the ones of the Californian/Mediterranean group, which clustered at the right of the first coordinate, suggests a loss of diversity from old European to Californian modern cultivars, as showed previously [21]. The proximity of modern French cultivars such as ‘Charlotte’ or ‘Cirafine’ to old European cultivars highlights the presence of old European germplasm, e.g. ‘Hummi Gento’ (from Netherland) or ‘Red Gaunlet’ (from UK) in their pedigree.

### Analysis of genetic diversity highlighted the pedigree in strawberry

Results obtained using the DArT data set were highly consistent throughout the three statistical tools used in this work and with the geographical, historical and pedigree data of the samples. The groups clustered varieties genetically related and these groups were also highlighted using STRUCTURE and PCoA. As an example, the three French varieties ‘Charlotte’, ‘Cirafine’ and ‘Cijosée’ illustrate the relationship between genotypes, arranged in the same cluster with the variety ‘Mara des Bois’, their maternal parent. This is extensible to ‘Pajaro’, ‘Sweet Charlie’, ‘Betty’ and CF1116 or to genotypes from our segregating population, the parents ‘1392’ and ‘232’ and their progeny 93–04, 93–54, 93–85 and 93–88 (Fig 1). Interestingly, some genotypes were clearly close to one of their parents but far from the other. As an example, cv. Darselect, issued from the cross ‘Elsanta’ × ‘Parker’, is closely related to ‘Elsanta’ but not to ‘Parker’. This result could be due to a distribution of the markers favorable to one parent to the detriment of the other.

### Performance of DArT-derived SNP markers in linkage mapping

Using the DArTseq derived markers, we have been able to increase marker density of the ‘232’ × ‘1392’ map to one marker every 1.34 cM. While the map still contain several double crossover events that can be reduced eliminating conflicting markers in the future, it provides a useful tool for further analyses such as QTL mapping. As an example, the DArTseq-saturated ‘232’ × ‘1392’ map has already been used for the identification of *FaFAD1* as a gene necessary for peach flavor in strawberry [81]. The length of the map, 2,490 cM, is slightly larger than previously published maps, in which total map lengths covered 2,050 to 2,364 cM [45,46,52,75,76]. Increasing the number of markers to more than 2,000 has resulted in extending the mapped regions of the octoploid genome and therefore to increase the length of the genetic map. However, taking into account the length of the ‘Holiday’ × ‘Korona’ recently published saturated map [52], which was only 2,050 cM, much larger increases in size could likely be due to genotyping errors rather than to such an increase in the represented genomic regions. Despite the high number of markers used for mapping, a total of 33 linkage groups (LG) were obtained, 5 more than the expected 28 strawberry chromosomes. We interpret this as a consequence of the close relationship between the parental lines, both with Californian pedigree (Table 1; Fig 1; Fig 2) as well as because of low heterozygosity especially for ‘1392’. Most probably because of this, several LGs were enriched in markers derived from one of the parental lines. Low heterozygosity in the cultivated strawberry has been described previously [46,52,76]. In the comparative genetic mapping between octoploid and diploid strawberry based on 51 SSRs, an average of 2.4 alleles per SSR was observed, which was lower than the 8 expected alleles in a situation of 100% heterozygosity [76]. In the ‘Holiday’ × ‘Korona’ linkage map, same chromosomal regions were homozygous based on SSR haplotype [46] and SNPs [52].

The high number of LGs detected for HG VI was surprising taking into account the number of markers used in this study. This could be a consequence of having the lowest number of polymorphic markers while being the largest chromosome in the diploid reference genome (Table B in S1 File). Similarly, 16 LGs from 5 different parental maps were used to produce the integrated LG 6A in the work of Isobe and collaborators [45] and more than four LGs belonging to HG VI were obtained in the DA × MO and ‘Sonata’ × ‘Babette’ maps [56,82]. One plausible explanation is that large regions of homozygosity that hamper linkage between adjacent markers are present in at least one of the LGs belonging to HG VI.

Intrachromosome rearrangements in the developed octoploid map compared to the reference diploid genome were abundant (Fig 5; S1 Fig) but the majority of those involving large genomic regions have been previously reported, indicating that they are real differences with the *F*. *vesca* genome. As an example, the same inversion or rearrangements in HG I and III compared to the *F*. *vesca* genome were detected in the RG × H map [75]. Similarly, an inversion in the distal part of pseudochromosome 2 compared to the HG II of octoploid strawberry was described in the ‘Holiday’ × ‘Korona’ map [46]. These authors also noticed an inversion that occurred in only one of the 4 homoeologous LGs, their LG2D. Increasing the density of the ‘232’ × ‘1392’ map resulted in the identification of the same inversion, that spans most of the length of LG II-2, indicating that this LG corresponds to LG2D in the ‘Holiday’ × ‘Korona’ map. Furthermore, this same inversion was detected in LG II-B1 of both octoploid progenitors of cultivated strawberry [57]. Octoploid strawberry sub-genome B1 is more similar to *F*. *iinumae* than to *F*. *vesca*, two ancestors considered to contribute to the sub-genomes of the octoploid *Fragaria* species [55,57]. Future comparisons with the *F*. *iinumae* genome could clarify whether this inversion was already present in a *F*. *innumae*-like ancestor or occurred later in only one of the sub-genomes of octoploid species. Other differences in marker position involved only one or two markers that were positioned far away such as those identified in HG VI and VII (S1 Fig). Since they were detected in more than one LG of each HG, these discrepancies could be explained as putative errors in the genome assemble of *F*. *vesca* or likely as the result of translocation or transpositions due to the action of transposable elements [57]. Overall, our results demonstrate the usefulness of DArTseq derived SNPs for genetic mapping in octoploid strawberry and for identifying rearrangements in the genome of the polyploid cultivated strawberry compared to the relative diploid species.

## Conclusion

In this work we report the development of two DArT marker platforms for high-throughput genotyping in the octoploid strawberry. The newly developed DArT platforms generated in this study demonstrated robust efficiency in the analysis of genetic diversity and structure of a diverse set of strawberry cultivars, and in increasing marker density in linkage maps. These newly developed marker systems complement the Axiom® IStraw90^®^ array developed previously for octoploid strawberry and overcome some of its current limitations. The availability of efficient genotyping for strawberry will enable better germplasm characterization and assist the identification of genes underlying QTLs linked to important agronomical traits.

## Supporting Information

S1 Fig Comparison between the ‘232’ × ‘1392’ octoploid linkage map (in red) and the diploid physical map based in the genome assembly of Tennessen et al., 2014 (in green). Rearrangements are highlighted.
(PDF)

S1 File Table A, List of markers mapped in the ‘232’ × ‘1392’ population, detailing adjacent sequence for DArTseq SNPs, quality scores, position in the octoploid map and in the diploid genome assembly of Tennessen et al. (2014), and genotypes in each progeny. Table B, Distribution of mapped markers in the ‘232’ × ‘1392’ map. (XLSX)
