# Supplementary material for: Diversity Arrays Technology (DArT) Marker Platforms for Diversity Analysis and Linkage Mapping in a Complex Crop, the Octoploid Cultivated Strawberry (Fragaria × ananassa)
Source: PLoS One. 2015 Dec 16;10(12):e0144960. doi: 10.1371/journal.pone.0144960 (PMC4682937; doi:10.1371/journal.pone.0144960)

Homoeology group 1

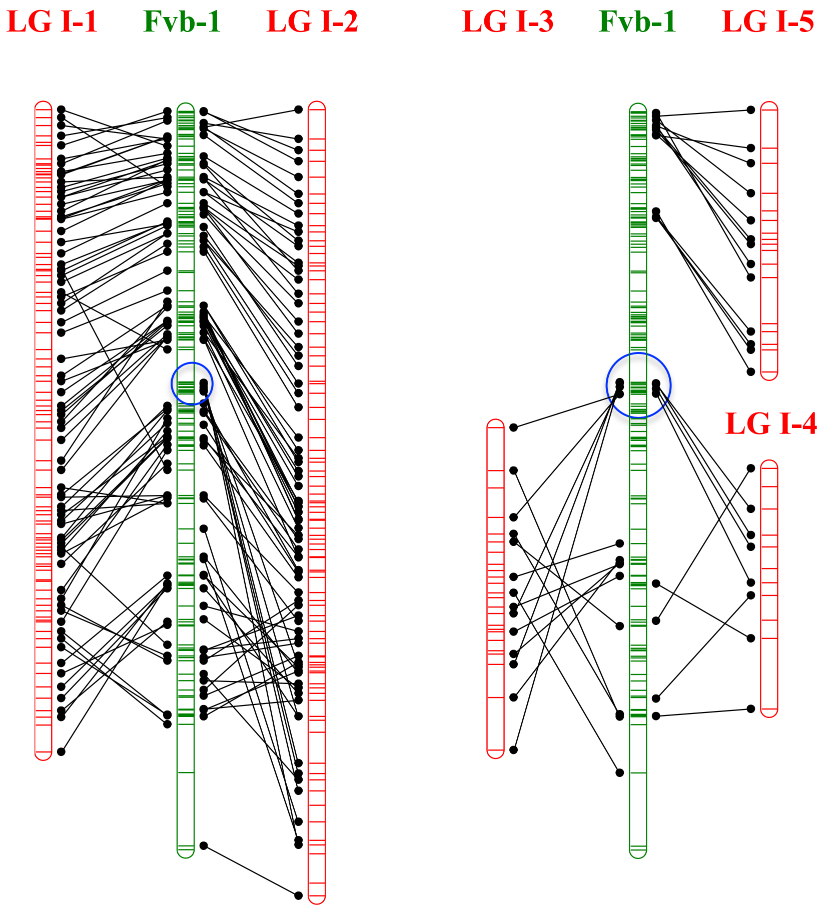

Supplementary Fig. S1. Comparison between the 232 x 1392 octoploid linkage map (in red) and the diploid physical map based in the genome assembly of Tennesse et al., 2014 (in green). Rearrangements are highlighted.

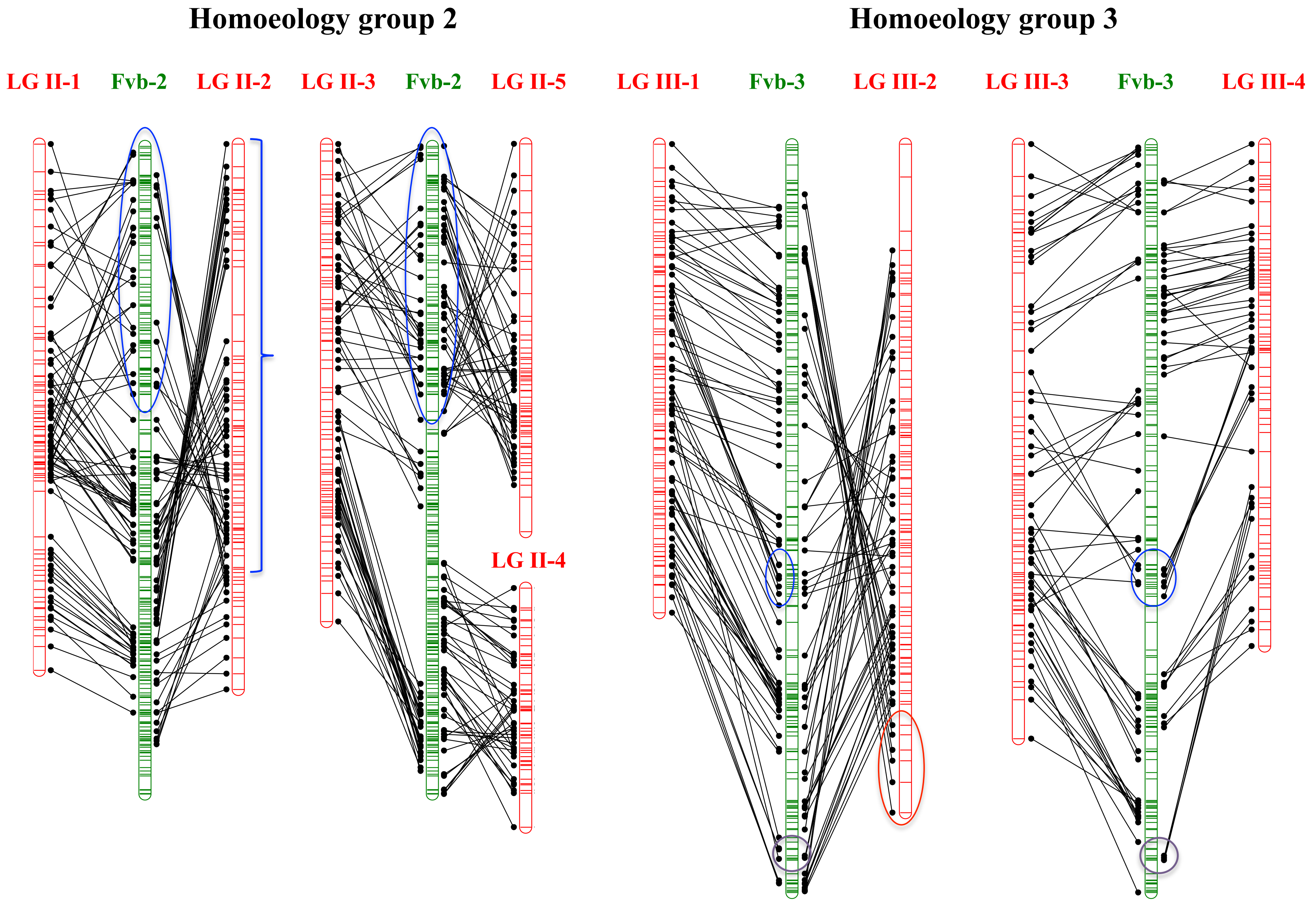
Homoeology group 2
Homoeology group 3
LG II-1
Fvb-2
LG II-2
LG II-3
Fvb-2
LG II-5
LG II-4
LG III-1
Fvb-3
LG III-2
LG III-3
Fvb-3
LG III-4

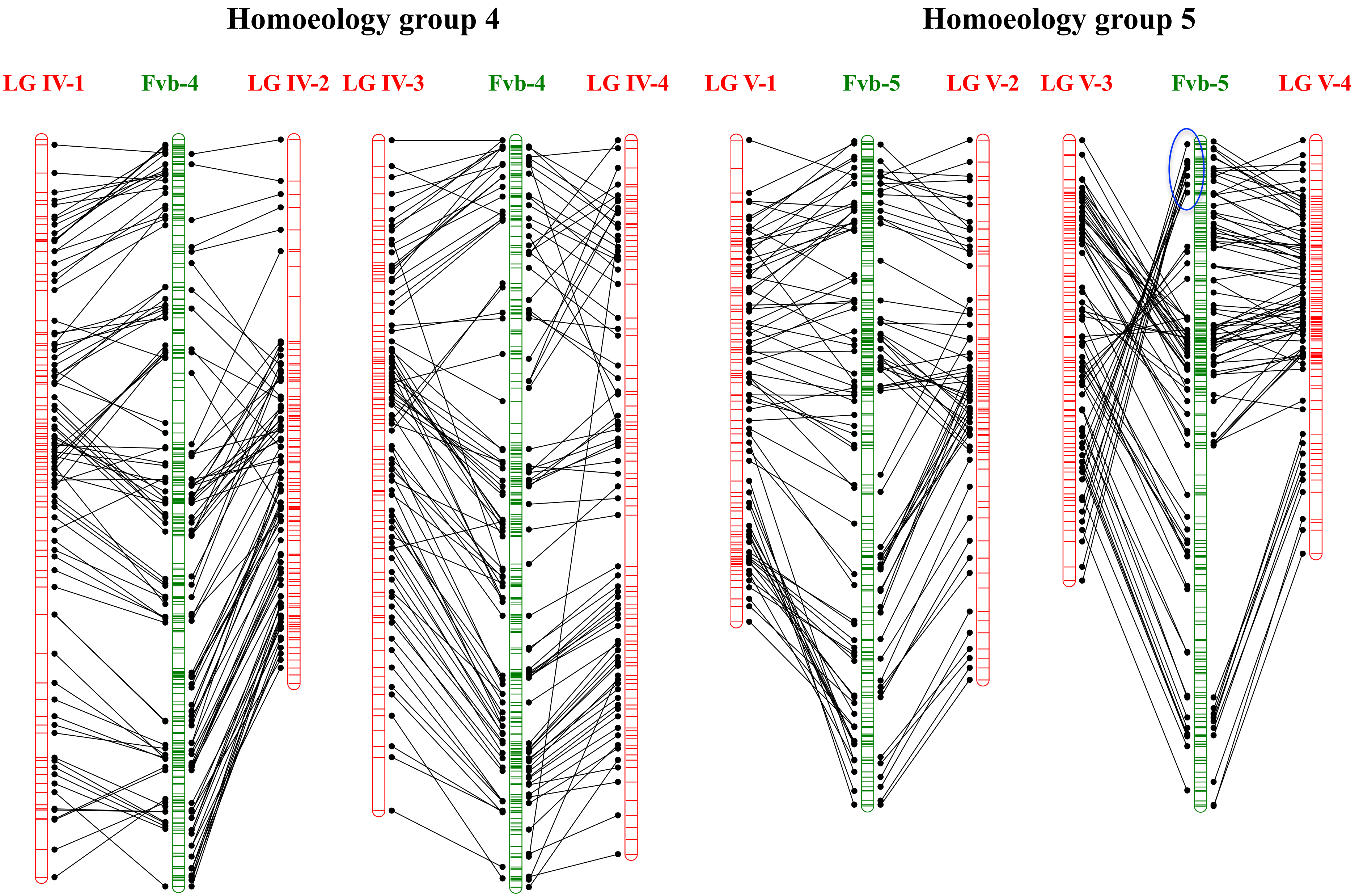

Homoeology group 4
Homoeology group 5
LG IV-1
Fvb-4
LG IV-2
LG IV-3
Fvb-4
LG IV-4
LG V-1
Fvb-5
LG V-2
LG V-3
Fvb-5
LG V-4

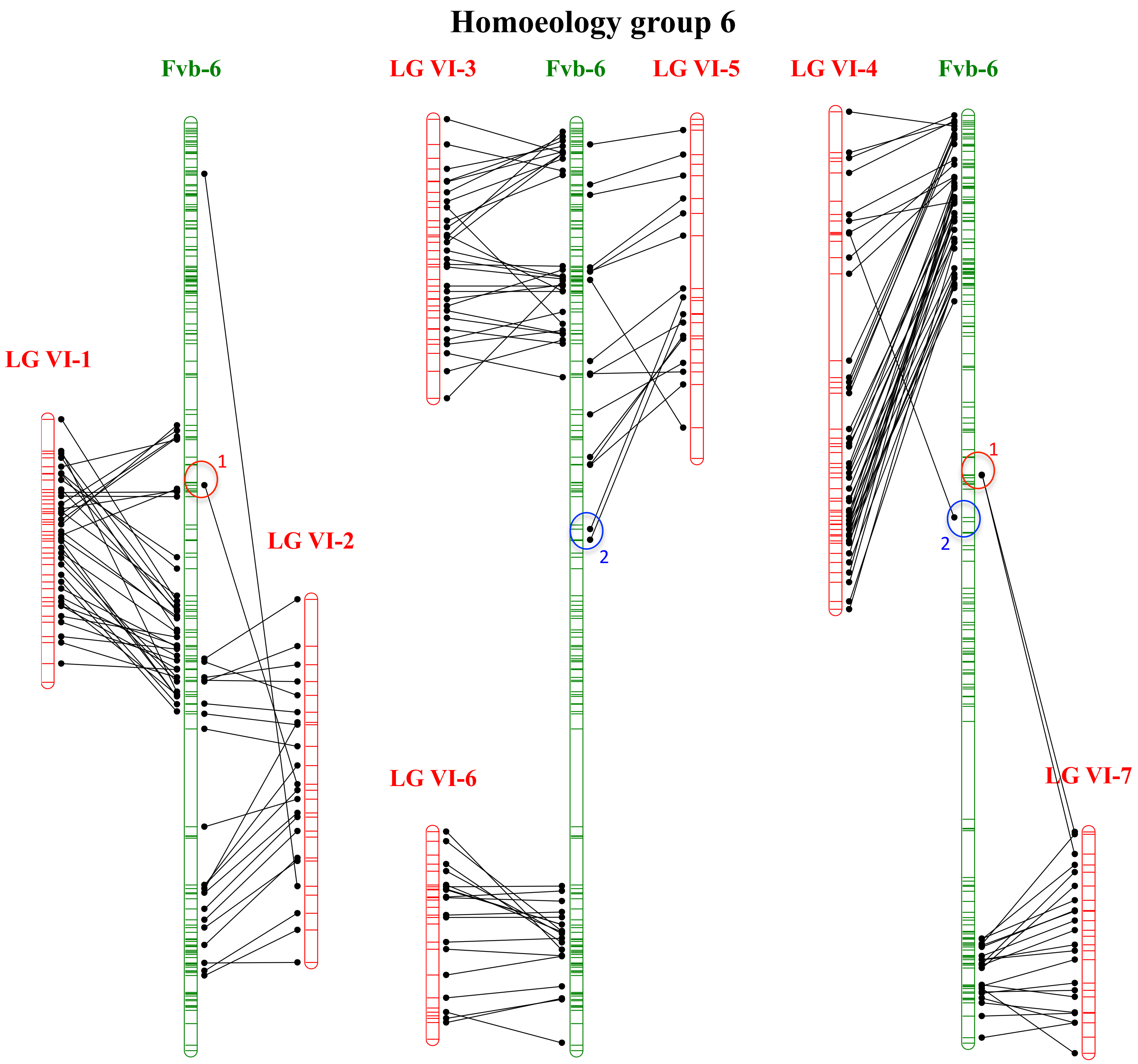

Homoeology group 6
Fvb-6
LG VI-3
Fvb-6
LG VI-5
LG VI-4
Fvb-6
LG VI-1
1
LG VI-2
2
1
2
LG VI-6
LG VI-7

Homoeology group 7

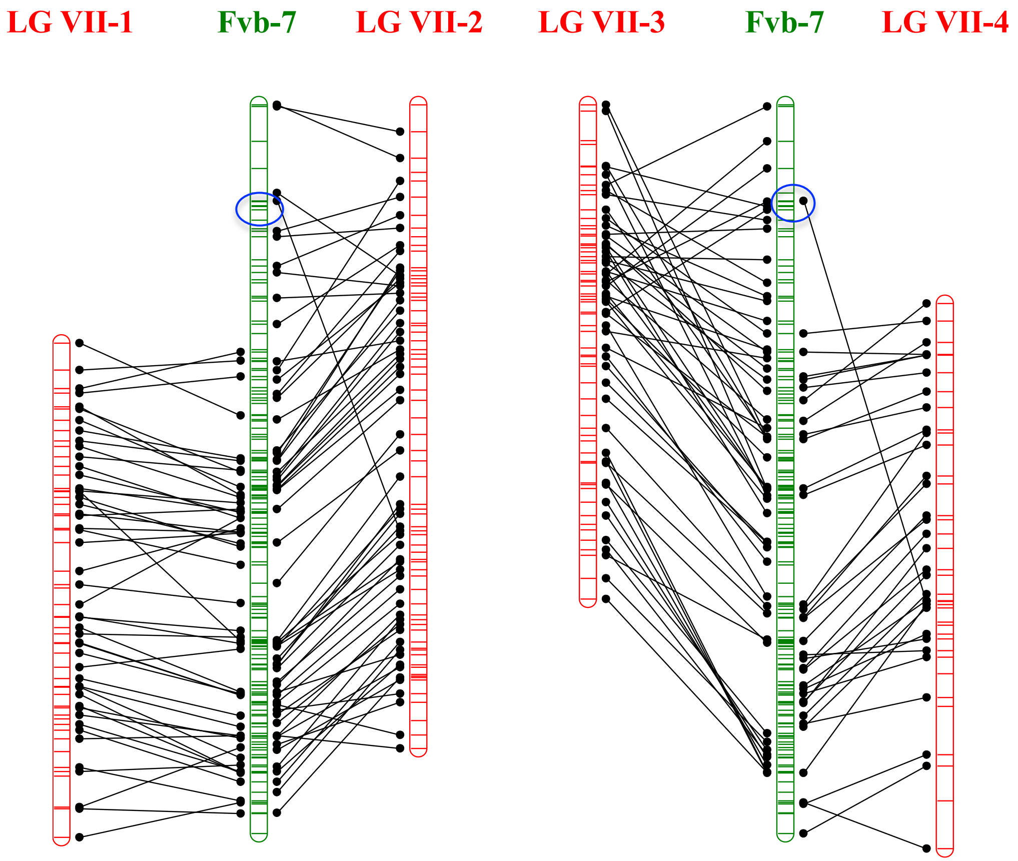

Supplement: S1 Fig — Rearrangements are highlighted. (PDF) [file pone.0144960.s001.pdf]
